# Supplementary material for: Empirical methods for controlling false positives and estimating confidence in ChIP-Seq peaks
Source: BMC Bioinformatics. 2008 Dec 5;9:523. doi: 10.1186/1471-2105-9-523 (PMC2628906; doi:10.1186/1471-2105-9-523)
Supplement: Additional File 1 — A variety of html documents from the USeq web site detailing the available applications, their best usage, output file type descriptions, command line menus, etc. [file 1471-2105-9-523-S1.zip › USeqUserGuides/JavaDoc.zip!/JavaDoc/bioroot/oligo/OligoBase.html]

OligoBase


|  |  |  |
| --- | --- | --- |
| **Overview** \| **Package** \| **Class** \| **Use** \| **Tree** \| **Deprecated** \| **Index** \| **Help** | |  |
| **PREV CLASS**   **NEXT CLASS** | **FRAMES**    **NO FRAMES**     **All Classes** |  |
| SUMMARY: NESTED \| FIELD \| CONSTR \| METHOD | DETAIL: FIELD \| CONSTR \| METHOD |  |


---


## bioroot.oligo Class OligoBase

```
java.lang.Object
  javax.servlet.GenericServlet
      javax.servlet.http.HttpServlet
          bioroot.oligo.OligoBase
```

**All Implemented Interfaces:**: java.io.Serializable, javax.servlet.Servlet, javax.servlet.ServletConfig

---

``` public class OligoBase extends javax.servlet.http.HttpServlet ```

**Author:**
:   nix
    Simple Oligo page, should use jsp. Contains static methods for oligos

**See Also:**: Serialized Form

---

| **Field Summary** | |
| --- | --- |
| `static Enzyme[]` | `ENZYMES` |
| `static java.lang.String[]` | `searchHeadings`             Use to make a hash of column heading in which to search, name then the actual search column name. |
| `static java.lang.String[]` | `stringFeatures`             Use to make a default oligo Feature[]. |


**Constructor Summary**

`OligoBase()`


| **Method Summary** | |
| --- | --- |
| `void` | `doGet(javax.servlet.http.HttpServletRequest request, javax.servlet.http.HttpServletResponse response)` |
| `void` | `doPost(javax.servlet.http.HttpServletRequest request, javax.servlet.http.HttpServletResponse response)` |
| `static java.lang.String` | `fetchConcentrationGroup(OligoBean ob)`             Returns a concentration unit |
| `static java.lang.String` | `getOligoForm(OligoBean ob, DBUtil bioRoot, UserBean userBean)`             Returns an oligo form filled it with the values in the oligo bean. |
| `static java.lang.String` | `getOligoReport(OligoBean ob, DBUtil bioRoot, UserBean userBean, boolean commonForm)`             Returns an oligo report. |
| `static java.lang.String[]` | `getSearchColumnNames()`             Returns a list of the search column names used in an SQL statment , ie Oligo.name, 1/2 the searchHash. |
| `static java.util.LinkedHashMap` | `getSearchHash()` |
| `static java.lang.String[]` | `getSearchNames()` |
| `static OligoBean[]` | `reverseOligoBeans(OligoBean[] beans)`             Reverses the order of an array of OligoBean. |

**Methods inherited from class javax.servlet.http.HttpServlet**

`service`

**Methods inherited from class javax.servlet.GenericServlet**

`destroy, getInitParameter, getInitParameterNames, getServletConfig, getServletContext, getServletInfo, getServletName, init, init, log, log`

**Methods inherited from class java.lang.Object**

`equals, getClass, hashCode, notify, notifyAll, toString, wait, wait, wait`

**Field Detail**

### ENZYMES

```
public static final Enzyme[] ENZYMES
```

---


### stringFeatures

```
public static java.lang.String[] stringFeatures
```

:   Use to make a default oligo Feature[]. Watch order of trues, must be on top

---


### searchHeadings

```
public static final java.lang.String[] searchHeadings
```

:   Use to make a hash of column heading in which to search, name then the actual search column name.


**Constructor Detail**

### OligoBase

```
public OligoBase()
```


**Method Detail**

### getSearchHash

```
public static java.util.LinkedHashMap getSearchHash()
```

---


### getSearchNames

```
public static java.lang.String[] getSearchNames()
```

---


### getSearchColumnNames

```
public static java.lang.String[] getSearchColumnNames()
```

:   Returns a list of the search column names used in an SQL statment , ie Oligo.name, 1/2 the searchHash.

---


### doGet

```
public void doGet(javax.servlet.http.HttpServletRequest request,
                  javax.servlet.http.HttpServletResponse response)
           throws javax.servlet.ServletException,
                  java.io.IOException
```

:   **Overrides:**: `doGet` in class `javax.servlet.http.HttpServlet`
:   **Throws:**: `javax.servlet.ServletException`: `java.io.IOException`

---


### doPost

```
public void doPost(javax.servlet.http.HttpServletRequest request,
                   javax.servlet.http.HttpServletResponse response)
            throws javax.servlet.ServletException,
                   java.io.IOException
```

:   **Overrides:**: `doPost` in class `javax.servlet.http.HttpServlet`
:   **Throws:**: `javax.servlet.ServletException`: `java.io.IOException`

---


### reverseOligoBeans

```
public static OligoBean[] reverseOligoBeans(OligoBean[] beans)
```

:   Reverses the order of an array of OligoBean.

---


### getOligoReport

```
public static java.lang.String getOligoReport(OligoBean ob,
                                              DBUtil bioRoot,
                                              UserBean userBean,
                                              boolean commonForm)
```

:   Returns an oligo report.

---


### getOligoForm

```
public static java.lang.String getOligoForm(OligoBean ob,
                                            DBUtil bioRoot,
                                            UserBean userBean)
```

:   Returns an oligo form filled it with the values in the oligo bean. Must add a form entry. ie
    + "

    " and start and stop HTML stuff

---


### fetchConcentrationGroup

```
public static java.lang.String fetchConcentrationGroup(OligoBean ob)
```

:   Returns a concentration unit


---


|  |  |  |
| --- | --- | --- |
| **Overview** \| **Package** \| **Class** \| **Use** \| **Tree** \| **Deprecated** \| **Index** \| **Help** | |  |
| **PREV CLASS**   **NEXT CLASS** | **FRAMES**    **NO FRAMES**     **All Classes** |  |
| SUMMARY: NESTED \| FIELD \| CONSTR \| METHOD | DETAIL: FIELD \| CONSTR \| METHOD |  |


---
